# Proteomic analysis of colony morphology variants of *Burkholderia pseudomallei* defines a role for the arginine deiminase system in bacterial survival

**DOI:** 10.1016/j.jprot.2011.10.015

**Published:** 2012-01-04

**Authors:** Narisara Chantratita, Sarunporn Tandhavanant, Chanthiwa Wikraiphat, Lily A. Trunck, Drew A. Rholl, Aunchalee Thanwisai, Natnaree Saiprom, Direk Limmathurotsakul, Sunee Korbsrisate, Nicholas P.J. Day, Herbert P. Schweizer, Sharon J. Peacock

**Affiliations:** a Department of Microbiology and Immunology, Faculty of Tropical Medicine, Mahidol University, Bangkok, Thailand; b Mahidol-Oxford Tropical Medicine Research Unit, Faculty of Tropical Medicine, Mahidol University, Bangkok, Thailand; c Department of Microbiology, Immunology and Pathology, Rocky Mountain Regional Center of Excellence for Biodefense and Emerging Infectious Diseases Research, Colorado State University, Fort Collins, Colorado, United States; d Department of Tropical Hygiene, Faculty of Tropical Medicine, Mahidol University, Bangkok, Thailand; e Department of Immunology, Faculty of Medicine Siriraj Hospital, Mahidol University, Bangkok, Thailand; f Centre for Tropical Medicine, Nuffield Department of Clinical Medicine, University of Oxford, Oxford, UK; g Department of Medicine, University of Cambridge, Addenbrooke's Hospital, Cambridge, UK

**Keywords:** Melioidosis, *Burkholderia pseudomallei*, Arginine deiminase system, Proteomic analysis, Colony variation

## Abstract

Colony morphology variation of *Burkholderia pseudomallei* is a notable feature of a proportion of primary clinical cultures from patients with melioidosis. Here, we examined the hypothesis that colony morphology switching results in phenotypic changes associated with enhanced survival under adverse conditions. We generated isogenic colony morphology types II and III from *B. pseudomallei* strain 153 type I, and compared their protein expression profiles using 2D gel electrophoresis. Numerous proteins were differentially expressed, the most prominent of which were flagellin, arginine deiminase (AD) and carbamate kinase (CK), which were over-expressed in isogenic types II and III compared with parental type I. AD and CK (encoded by *arcA* and *arcC*) are components of the arginine deiminase system (ADS) which facilitates acid tolerance. Reverse transcriptase PCR of *arcA* and *arcC* mRNA expression confirmed the proteomic results. Transcripts of parental type I strain 153 *arcA* and *arcC* were increased in the presence of arginine, in a low oxygen concentration and in acid. Comparison of wild type with *arcA* and *arcC* defective mutants demonstrated that the *B. pseudomallei* ADS was associated with survival in acid, but did not appear to play a role in intracellular survival or replication within the mouse macrophage cell line J774A.1. These data provide novel insights into proteomic alterations that occur during the complex process of morphotype switching, and lend support to the idea that this is associated with a fitness advantage in vivo.

## Graphical abstract

**Figure:**
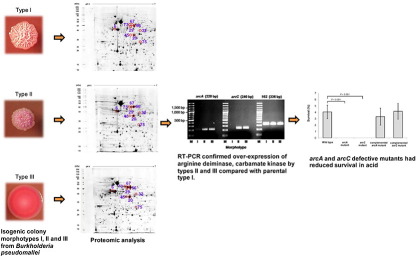

**Highlights:**

► *B. pseudomallei* undergoes morphotypic switching in response to stress. ► Numerous proteins were differentially expressed by isogenic morphotypes. ► Types II and III over-expressed arginine deiminase (ArcA) and carbamate kinase (ArcC). ► Exposure of parental type I to acid led to increased transcription of *arcA* and *arcC*. ► *arcA* and *arcC* defective mutants had reduced survival in acid.

## 1 Introduction

*Burkholderia pseudomallei* is an environmental Gram-negative bacillus and the cause of melioidosis, a life-threatening infection endemic to Southeast Asia and Northern Australia [1,2]. Melioidosis presents with a broad clinical spectrum ranging from mild localized infection to rapidly fatal septicemia. The more severe end of the spectrum is common, and infection is associated with a mortality rate of around 40% in northeast Thailand where the majority of cases are reported. Notable features relating to treatment include a slow response to antimicrobial drugs, the need for prolonged antimicrobial therapy, and relapse despite apparently adequate antibiotic treatment [1–3].

The colony morphology of *B. pseudomallei* on Ashdown agar (a selective medium used by laboratories that isolate this organism on a regular basis) is usually purple, wrinkled, and likened to a cornflower head. This appearance has been classified as type I, with the description of a further six less common types (types II to VII) during a study of primary plates from diagnostic cultures [4]. This observation can be explained by the process of colony morphology switching, a reversible event in which a given colony type can switch to an alternative type. Previously, we demonstrated that reversible alterations in *B. pseudomallei* morphotype were associated with reproducible and reversible changes in bacterial length, production of extracellular enzymes, biofilm formation, and flagella production [4]. We have proposed that such changes reflect a mechanism by which *B. pseudomallei* can survive unfavorable conditions. Although most likely to have evolved in response to survival in the environment, this may have important implications for host–pathogen interactions and persistence in the human host following infection.

Evidence for the role of *B. pseudomallei* colony morphology switching in disease comes from both animal and in vitro models. In an experimental mouse model, type II appeared to become adapted for persistence, and type III was associated with switching to type I or II [4]. Colony morphology switching has also been reported to occur after uptake of *B. pseudomallei* by macrophages in vitro, with a difference in the rate of bacterial replication between the different morphotypes after uptake [4,5].

The objective of this study was to expand on the number of proteins known to undergo differential expression during colony morphology switching, and to provide proof of concept that one or more of these proteins are involved in survival under adverse conditions. We generated isogenic types II and III from parental type I using starvation conditions, and compared protein expression by these three types using 2D gel electrophoresis. Numerous proteins were observed to be differentially expressed, the most prominent of which were flagellin, arginine deiminase (AD) and carbamate kinase (CK), which were over-expressed in isogenic types II and III compared with parental type I. AD and CK (encoded by *arcA* and *arcC*) are components of the arginine deiminase system (ADS) that catalyzes the metabolism of arginine to ornithine, ammonia, and CO_2_ with the generation of ATP [6]. We hypothesized that the ADS facilitated adaptation and survival in adverse environments. We examined this question using transcript analysis of *arcA* and *arcC*, and by comparing survival of a parental strain versus mutants defective in each of these two genes after exposure to acid and following uptake by macrophages in vitro.

## 2 Materials and methods

### 2.1 *B. pseudomallei* strains and isolation of isogenic morphotypes

*B. pseudomallei* strain 153 type I was isolated from the blood of a patient admitted to Sappasithiprasong hospital, Ubon Ratchathani, Thailand in 2002. Two isogenic variants defined on the basis of colony morphology appearance as types II and III were obtained from the parental type I isolate using nutritional limitation, as described previously [4]. In brief, a single colony of type I was cultured in trypticase soy broth (TSB) at 37 °C under static conditions in air for 21 days. Serial dilutions were spread plated onto Ashdown agar, incubated at 37 °C in air for 4 days and the colony morphologies classified using a morphotyping algorithm described previously [4]. Isogenic types II and III were harvested and stored in TSB containing 20% glycerol at − 80 °C, (a condition under which colony morphotype is stable) until use. The three isogenic types had identical banding patterns by pulsed-field gel electrophoresis (PFGE) using *Spe*I restriction enzyme digestion [4].

### 2.2 Protein extraction

Protein extraction was performed on two separate occasions for the three isogenic morphotypes. A single colony of *B. pseudomallei* picked from Ashdown agar was cultured in 100 ml of Luria-Bertani (LB) broth at 37 °C with shaking at 200 rpm for 18 h. Bacteria were centrifuged at 4500 × *g* for 30 min at 4 °C, washed once with cold PBS and then resuspended in 1 ml of a cold lysis buffer containing 5 mM EDTA and 1 mM phenylmethylsulfonyl fluoride (PMSF) [7]. The suspension was sonicated on ice at 22% amplitude at 1 second pulse intervals for 3 min, and the lysate centrifuged at 14,000 × *g* for 3 min at 4 °C. The supernatant was collected and sterilized by passage through a 0.2 μm filter and stored at − 80 °C until use.

### 2.3 Two-dimensional (2D) gel electrophoresis

Protein samples were cleaned using the 2D clean-up kit (GE Healthcare Bio-Sciences), and the concentration determined with the 2D quantification kit (GE Healthcare Bio-Sciences) using BSA as a standard. Seven hundred micrograms of protein was mixed with rehydration buffer (8 M Urea, 2% CHAPS, 60 mM DTT, 0.5% IPG buffer pH 4–7 and 0.002% bromophenol blue) to a total volume of 340 μl per immobiline dry strip. A 18-cm IPG strip pH 4–7 was rehydrated with this sample solution for 12 h at room temperature. Isoelectric focusing (IEF) was performed using an Ettan IPGphor 3 isoelectric focusing system (GE Healthcare Bio-sciences) at 20 °C. The running steps were set as follows: step 1: 500 V for 1 h, step 2: 1000 V for 1 h, step 3: 8000 V for 3 h and step 4: 8000 V for 1.30 h. On completion, the strip was equilibrated in 5 ml SDS-PAGE loading buffer (50 mM Tris–HCl, pH 8.8, 6 M urea, 30 glycerol, 2% SDS and trace amount of bromophenol blue) containing 50 mg dithiothreitol (DTT) and 125 mg iodoacetamide (IAA) with continuous shaking for 15 min. The strip was then submerged in 1 × SDS electrophoresis running buffer (25 mM Tris–HCl, pH 8.3, 192 mM glycine, 0.1% SDS) and subjected to second dimensional separation using a 12.5% acrylamide gel. Protein separation was performed in a SE600 Ruby vertical electrophoresis unit (GE Healthcare Bio-Sciences) using a current/gel of 10 mA for 15 min and 20 mA until the dye front was 1 mm from the bottom of the gel. Protein spots were visualized with colloidal Coomassie blue G-250 stain (8% ammonium sulfate, 0.8% phosphoric acid, 0.08% Coomassie blue G-250 and 20% methanol). Gel images were captured using an Image Scanner II and LabScan software version 5.0 (GE Healthcare Bio-Sciences). The intensity of protein spots was initially compared by eye between isogenic types I vs. II and I vs. III, and those with visible differences analyzed further using ImageMaster 2D Platinum version 5.0 (GE Healthcare Bio-Sciences). Two protein preparations were examined for each type in independent experiments.

### 2.4 In-gel digestion and protein identification by matrix-assisted laser desorption/ionization time of flight mass spectrometry (MALDI-TOF MS)

Proteins of interest were picked from the gel using the Ettan Spot Handling Workstation (GE Healthcare Bio-Sciences). Gel plugs were washed twice with 100 μl 50 mM ambic/50% methanol, dehydrated in 100 μl 75% acetonitrile (ACN) for 10 min and then dried. Plugs were rehydrated in 20 mM ambic and digested with 10 μl of trypsin solution at 37 °C for 1 h. Peptides were extracted twice with 60 μl of 50% ACN/0.1% trifluoroacetic acid (TFA) for 20 min and with 40 μl of 50% ACN/0.1% TFA for 20 min, then the two extracts were pooled and dried. Peptides were resuspended in 3 μl of 50% ACN/0.1% TFA, then mixed with equal volumes of matrix solution (α-cyano-4-hydroxy-cinnamic acid in 50%ACN, 0.1% TFA and 2% w/v ammonium citrate) and applied onto a target plate (MTP 384 polished steel TF). All samples were analyzed using an Autoflex MALDI-TOF MS (Bruker Daltonik, Bremen, Germany) at the BioService Unit (BSU), National Science and Technology Development Agency, Pathumthani, Thailand. Protein mass fingerprints were searched for protein identity and sequence using the MASCOT search engine (http://www.matrixscience.com). Protein identification was achieved by a protein blast search using the NCBI protein database. (http://blast.ncbi.nlm.nih.gov/Blast.cgi). The cellular localization of proteins was predicted using PSORTb version 3.0.2 (www.psort.org/psortb).

### 2.5 Reverse transcriptase PCR for *arcA* and *arcC* gene expression

**Fig. 1:**
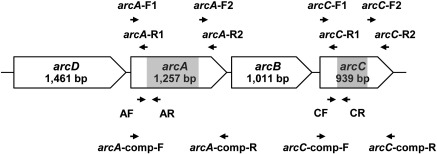
Gene organization of the arginine deiminase system (ADS) of *B. pseudomallei*. Primer binding sites are indicated by arrows. Highlights indicate the extent of DNA deleted in the *arcA* and *arcC* mutants.

*B. pseudomallei* cells were harvested and RNA extracted using Trizol reagent (Invitrogen). RNA concentration was measured using the Nanodrop method (Thermo Scientific). Primers were designed for *arcA* (BPSL1743) and *arcC* (BPSL1745) using Primer-BLAST (http://www.ncbi.nlm.nih.gov/tools/primer-blast). Primer sequences are shown in Table 1, and primer binding sites in *arcA* and *arcC* are indicated in Fig. 1. One-step reverse transcriptase (RT-PCR) was performed using 1 μg RNA and a Superscript III One-step RT-PCR system (Invitrogen) with forward primer AF and reverse primer AR for *arcA*, or forward primer CF and reverse primer CR for *arcC* (Fig. 1 and Table 1). The RT-PCR conditions were as follows: cDNA synthesis at 45 °C for 30 min; initial denaturation at 95 °C for 2 min; 35 cycles of denaturation at 94 °C for 15 s, annealing at 58 °C (*arcA*) or 64 °C (*arcC*) for 15 s, and extension at 72 °C for 15 s; and a final elongation step at 72 °C for 5 min. The positive control was RT-PCR for 16S rDNA amplification using primers Univ_16S_F and Univ_16S_R (Table 1). The negative controls were *arcA* and *arcC* reactions without reverse transcriptase (RT) enzyme. The amplified product was run on a 1.5% agarose gel, stained with ethidium bromide and visualized under UV light using GeneSnap V 6.08 (SynGene, Cambridge, England).

RT-PCR was used to determine *arcA* and *arcC* expression in late log phase by *B. pseudomallei* strain 153 isogenic types I, II and III. One colony of *B. pseudomallei* on Ashdown agar was inoculated into 5 ml LB broth and incubated at 37 °C with shaking at 200 rpm for 12 h. The culture was centrifuged at 12,000 rpm for 5 min, and RNA extracted from the pellet and quantified as described above.

RT-PCR was also used to determine *arcA* and *arcC* expression by *B. pseudomallei* strain 153 type I under a range of conditions in vitro. One colony of *B. pseudomallei* on Ashdown agar was inoculated into each of the following: (i) 5 ml LB broth at pH 7.4; (ii) LB broth at pH 4.0; or (iii) LB broth at pH 7.4 containing 50 mM l-arginine. Duplicate broths were prepared for 5 ml LB broth at pH 7.4, with one tube incubated at 37 °C in air with shaking at 200 rpm and the other incubated with a loosened cap at 37 °C under oxygen limiting conditions using an airtight plastic container (Oxoid) with an AnaeroPack-MicroAero system (MGC, Japan). After 12 h incubation, *B. pseudomallei* cells were harvested and RNA extracted and quantitated as before. 1 μg RNA was analyzed for each experiment, and all experiments were performed on 2 separate occasions.

### 2.6 Construction of *arcA* and *arcC* mutants and complemented strains

The fragment mutagenesis method described by Lopez et al. [8] was employed to generate *arcA* or *arcC* defective mutants in *B. pseudomallei* strain 153. *B. pseudomallei* K96243 *arcA* and *arcC* sequences were obtained from GenBank (accession number NC_006350, [9]). PCR primers corresponding to 5′ (*arcA*-F1 and *arcA*-R1) and 3′ fragments (*arcA*-F2 and *arcA*-R2) of *arcA* (BPSL1743) and 5′ (*arcC*-F1 and *arcC*-R1) and 3′ fragments (*arcC*-F2 and *arcC*-R2) of *arcC* (BPSL1745) were designed using Primer-BLAST (http://www.ncbi.nlm.nih.gov/tools/primer-blast). The primer sequences and product sizes are shown in Table 1. Forward primers amplifying the 3′ fragments of these genes (*arcA*-F2 and *arcC*-F2) were designed to have an oligonucleotide tail homologous to the 3′ ends of 5′ fragments. DNA was extracted from *B. pseudomallei* parental type I strain 153 using a High Pure PCR template preparation kit (Roche) after overnight culture in LB broth at 37 °C with shaking at 200 rpm. The 5′ and 3′ fragments of each gene were joined by PCR using *arcA*-F1 and *arcA*-R2 or *arcC*-F1 and *arcC*-R2 primers, which was facilitated by a tail on the 3′ forward primer to give a new PCR product with a deletion in the region between *arcA*-R1 and *arcA*-F2 or between *arcC*-R1 and *arcC*-F2. These mutant constructs were cloned into pGEM®_T Easy and transformed into *Escherichia coli* DH5α. White colonies were selected using β-galactosidase indicator medium containing 50 μg/ml 5-bromo-4-chloro-3-indolyl-β-d-galactopyranoside (X-Gal) (Promega) plates containing 100 μg/ml ampicillin. Colonies containing the desired plasmids were analyzed by PCR using primers flanking the mutant alleles (*arcA*-F1 and *arcA*-R2 or *arcC*-F1 and *arcC*-R2). The products were checked for correct size by agarose gel electrophoresis and verified by DNA sequencing.

The unmarked knockout cassettes assembled by PCR and containing deletions in the *arcA* and *arcC* genes were cloned into the non-replicative plasmid, pEXKm5 [8]. The pEXKm5-mutant allele constructs were transformed into *E. coli* DH5α. Plasmids were extracted and checked by PCR with primers *arcA*-F1 and *arcA*-R2 or *arcC*-F1 and *arcC*-R2 for the correct product sizes of target gene constructs. The pEXKm5-mutant plasmids were then transformed into *E. coli* RHO3 and delivered to the host *B. pseudomallei* strain 153 by conjugation as previously described [8], resulting in integration of the allelic replacement construct into the *B. pseudomallei* chromosome by homologous recombination between cloned and chromosomal sequences. The merodiploid clones visualized as blue colonies on LB agar containing 1000 μg/ml kanamycin and 50 μg/ml 5-bromo-4-chloro-3-indolyl-β-d-glucuronide (X-Gluc) were selected for PCR with primers flanking the mutant alleles (*arcA*-F1 and *arcA*-R2 or *arcC*-F1 and *arcC*-R2) (Fig. 1 and Table 1).

For merodiploid resolution, clones were streaked onto yeast extract tryptone (YT) agar (Yeast extract and tryptone, BD; agar, Oxoid) containing 15% sucrose and 50 μg/ml X-Gluc and incubated at 25 °C for 72 h. White colonies growing on X-Gluc-containing medium (YT-sucrose-X-Gluc plate) were selected and purified by streaking on the same medium and incubating as described above. Merodiploid resolution leads to formation of either a wild type or a mutant strain, which were distinguished by PCR using primer sets flanking the mutant deletion alleles primers (*arcA*-F1 and *arcA*-R2 or *arcC*-F1 and *arcC*-R2) and the *oriT* pEXKm5 plasmid backbone sequences. In contrast to BP153, *arcA* and *arcC* mutants yielded smaller DNA fragments for the deleted region and did not yield *oriT* associated PCR products. Both mutants and wild type strain did not grow on LB agar with 1000 μg/ml kanamycin.

Complemented strains were constructed using the same pEXKm5-based allele replacement approach. The forward and reverse primers corresponding to the relevant regions of the genome sequences were designed and their sequences and locations are shown in Table 1 and Fig. 1, respectively (*arcA*-comp-F and *arcA*-comp-R or *arcC*-comp-F and *arcC*-comp-R primers). The PCR amplicons (*arcA* 1261 bp and *arcC* 996 bp) contained wild type *B. pseudomallei* strain 153 *arcA* and *arcC* sequences plus upstream and downstream sequences. The constructs were cloned into pEXKm5 [8], transformed into *E. coli* RHO3 and delivered to *B. pseudomallei* mutants by conjugation, resulting in merodiploid formation. Sucrose selection was employed for merodiploid resolution resulting in generation of wild type sequences containing strains as well as strains that maintained the deletion alleles. PCRs were performed with primers flanking deleted alleles to screen for strains that had the mutant alleles replaced with wild type sequences. PCR with *oriT* specific primers was used to demonstrate the absence of pEXKm5 plasmid backbone.

Gene deletions were verified by RT-PCR following gene induction using l-arginine and oxygen limitation. *B. pseudomallei* was cultured on LB agar containing 50 mM l-arginine at 37 °C for 24 h under oxygen limitation as described above. Bacteria were harvested, and the RNA extracted, quantified and analyzed as described above.

### 2.7 Growth curve analysis

Colonies of *B. pseudomallei* 153 wild type, *arcA* or *arcC* mutants were picked from Ashdown agar, suspended in sterile PBS and adjusted to an optical density (OD) at 600 nm to obtain bacterial suspensions of approximately 1 × 10^6^ cfu/ml. One hundred microlitres of this suspension was added to 10 ml of LB and incubated at 37 °C in air with shaking at 200 rpm for 24 h. At 1, 3, 6, 9, 12, 15 and 24 h a 500 μl aliquot of bacterial culture was removed for turbidity measurements at 600 nm.

### 2.8 Susceptibility of *B. pseudomallei* to acid

*B. pseudomallei* strain 153 type I (wild type), *arcA*, *arcC* mutants and complemented strains with restored genotypes were harvested from Columbia agar plates following incubation overnight 37 °C in air, then washed and resuspended in PBS. The suspension was adjusted to an OD at 600 nm to obtain a bacterial concentration of approximately 1 × 10^8^ cfu/ml. The number of bacteria in the inoculum was verified by plate count. One hundred microlitres of suspension was inoculated into 5 ml of LB broth pH 3.0 or 4.0 containing 50 mM arginine, and incubated at 37 °C with shaking at 200 rpm for 6 h. The numbers of viable bacteria were quantified by plating serial dilutions on Columbia agar plates. Percent survival was calculated by (number of viable bacteria × 100) / number of bacteria in starting inoculum. The experiment was performed in triplicate.

### 2.9 Growth of *B. pseudomallei* under oxygen limitation

*B. pseudomallei* wild type, *arcA* or *arcC* mutants grown overnight on LB agar were suspended in PBS, the bacterial cell counts adjusted to approximately 1 × 10^8^ cfu/ml and then diluted using 10-fold serial dilutions in PBS. Ten microlitres of bacterial suspension from each dilution was dropped in triplicate onto two sets of LB agar plates adjusted to pH 7.4. One set of plates were incubated at 37 °C in air, and the second set incubated at 37 °C in an AnaeroPack-MicroAero system as described above. The numbers of viable bacteria were determined at 48 h and compared with the bacterial count in the starting inoculum, and presented as bacterial survival (cfu/ml). The experiment was performed in triplicate on two separate occasions.

### 2.10 Interaction of *B. pseudomallei* with macrophages

A mouse macrophage cell line J774A.1 (ATCC TIB-67) was used in this study. Cells were maintained in Dulbecco's modified Eagle medium (DMEM, Invitrogen) supplemented with 10% fetal bovine serum (PAA Laboratories, Austria). The invasion assay was performed as previously described [4]. In brief, *B. pseudomallei* cells from an overnight culture of wild type, *arcA* or *arcC* mutants on Ashdown agar were suspended in PBS, the bacterial cell density adjusted using OD at 600 nm and then diluted in PBS. This was used to inoculate a 24-well plate with each well containing an overnight culture of 1 × 10^5^ J774A.1 cells to obtain a multiplicity of infection (MOI) of approximately 10 bacteria per cell. The MOI was verified by bacterial colony counting on Columbia agar. Infected J774A.1 cells were incubated at 37 °C in 5% CO_2_ for 2 h, the medium replaced with medium containing 250 μg/ml of kanamycin and then incubated for a further 2 h to kill external bacteria, before replacing with fresh medium containing 20 μg/ml of kanamycin. At the indicated time points, the cells were washed three times, lysed, and the cell lysates serially diluted in PBS and spread plated on Ashdown agar to obtain the bacterial count. The percentage of bacteria that were cell-associated was calculated by (number of associated bacteria × 100)/number of bacteria in the inoculum. The experiment was performed in triplicate on two separate occasions.

### 2.11 Statistical analysis

Statistical analysis was performed using the statistical program STATA version 11 (Stata Corp, College Station, TX, USA). Log transformation of continuous dependent variables was performed. Repeated measures ANOVA was used to compare continuous dependent variables between wild type, *arcA* and *arcC* mutants. *P* value for pairwise comparisons was adjusted using the Benjamini–Hochberg method [10]. Differences between wild type and the two mutants were considered to be statistically significant when the *P* value was ≤ 0.05.

## 3 Results

### 3.1 Proteomic analysis of *B. pseudomallei* strain 153 types I, II and III

Proteomic analysis of *B. pseudomallei* strain 153 revealed over 700 protein spots. Protein expression was compared between *B. pseudomallei* strain 153 types I versus II, and types I versus III using 2D Image master software. Only those proteins with a reproducible difference of at least 1.5 fold in spot intensity volume in two independent experiments are reported. Differential expression was observed for 19 protein spots (Table 2). Of these, 9 proteins showed increased expression in types II and III compared with type I, and 10 proteins had reduced expression in types II and III compared with type I (Table 2). No proteins were differentially expressed in only type II or type III.

These 19 proteins were identified and grouped by COG functional category (http://www.ncbi.nlm.nih.gov).

**Fig. 2:**
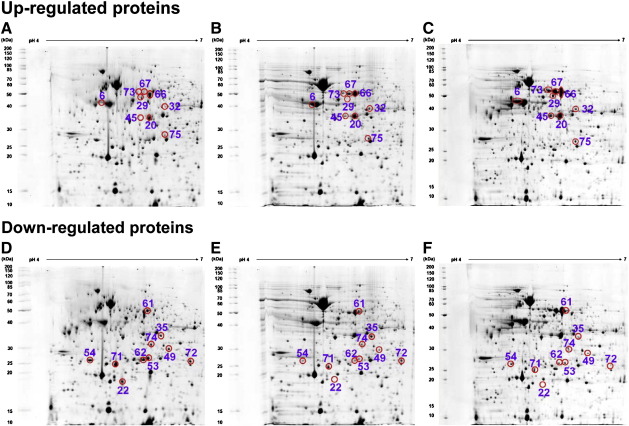
Proteomic profiles of *B. pseudomallei* strain 153 type I (A, D) and isogenic type II (B, E) and type III (C, F). Red circles are used to highlight up-regulated proteins (A, B and C) or down-regulated proteins (D, E and F) in types II and III compared with type I. Protein spot numbers relate to information provided in the text and Table 2.

Five of the 9 proteins with increased expression in types II and III were involved in amino acid transport and metabolism. Two protein spots (number 66 and 67, Fig. 2) were identified as arginine deiminase (AD), and a further two spots (number 20 and 45, Fig. 2) were identified as carbamate kinase (CK). These two proteins are part of the arginine deiminase system. The fifth was a hypothetical protein (spot 32) with similarity to a dehydrogenase of *Agrobacterium tumefaciens*. Other proteins with increased expression in types II and III were ferredoxin-NADP reductase (spot 75), UDP-glucose dehydrogenase (spot 73) and chaperone GroEL protein (spot 29) (Table 2). Flagellin (spot 6) was markedly over-expressed in types II and III (11 and 16 fold, respectively) (Table 2 and Fig. 2).

Of the 10 down-regulated proteins in types II and III, two had homology to succinyl-CoA (3-ketoacid-coenzyme A transferase subunit A (spot 53) and subunit B (spot 54), and two proteins were involved in energy production and conversion including inorganic pyrophosphatase (spot 22). Another 3 proteins were classified under transport and metabolism of amino acids, inorganic ions or secondary metabolites (Table 2). One protein was classified as peroxidase (spot 62), one was classified as a multifunctional protein identified as acetoacetyl-CoA reductase (spot 72), and the final protein was of unknown function coded by BPSL1549 (spot 71) (Table 2 and Fig. 2).

Prediction of sub-cellular organization of proteins using PSORTb demonstrated that almost all of these differentially expressed proteins were located in the cytoplasmic compartment of bacterial cells, with the exceptions of flagellin protein which is assembled in the cytoplasm and secreted across the cell wall to be anchored to the cell membrane, and UDP-glucose dehydrogenase which may be located in multiple sites. BPSL1549 is a hypothetical protein of unknown location.

In *Pseudomonas aeruginosa*, AD and CK are encoded by *arcA* and *arcC*[11]. Comparison of the genome of *B. pseudomallei* strain K96243 and *P. aeruginosa* strain ATCC 15692 using webACT (http://www.webact.org) demonstrated that the gene organization of these two species are similar, with *arcD*, *arcA*, *arcB* and *arcC* present in this gene order within an operon structure (Fig. 1). The amino acid identity between *B. pseudomallei* and *P. aeruginosa* was 79.4% for ArcA and 82.6% for ArcC. We noted that that there was no similarity between these two species in the region upstream of *arcD*, which in *B. pseudomallei* contained BPSL1741 encoding a hypothetical protein and in *P. aeruginosa* contains an ANR box transcription initiation site [11].

### 3.2 *arcA* and *arcC* expression by RT-PCR

**Fig. 3:**
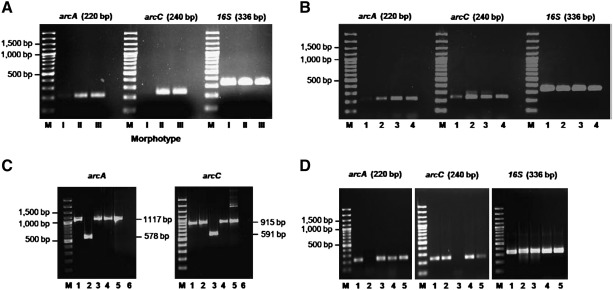
Reverse transcriptase PCR analysis of *arcA* and *arcC* (A) Transcript of *arcA*, *arcC* and 16S RNA (control) for *B. pseudomallei* strain 153 isogenic types I, II and III cultured in LB at 37 °C in air. M: 100–3000 bp ladder. (B) Transcript levels of *arcA*, *arcC* and 16S RNA for *B. pseudomallei* strain 153 type I (parental) cultured as follows: lane 1, LB broth pH 7.4; lane 2, LB broth pH 4.0; lane 3, LB broth containing 50 mM l-arginine, pH 7.4 incubated at 37 °C in air; lane 4, LB broth at pH 7.4 incubated at 37 °C under oxygen limiting conditions. M: 100–3000 bp ladder. (C) PCR analysis of *B. pseudomallei* strain 153 type I (parental), its *arcA* and *arcC* mutants and complemented strains cultured in LB at 37 °C in air. The sizes of DNA fragments amplified with primer sets *arcA*-F1 and *arcA*-R2 for *arcA* and *arcC*-F1 and *arcC*-R2 for *arcC* are indicated on the right of the respective panel. The larger fragments are products obtained with *B. pseudomallei* strain 153 templates and the smaller fragments are products obtained with *arcA* and *arcC* mutant templates. Lane 1, wild type; lane 2, *arcA* mutant; lane 3, *arcC* mutant; lane 4, *arcA* complemented strain; lane 5, *arcC* complemented strain; and lane 6, negative control. M: 100–3000 bp marker ladder. (D) RT-PCR analysis of *arcA*, *arcC* and 16 S rRNA transcript levels in *B. pseudomallei* strain 153 type I (parental), *arcA* and *arcC* defective mutants and complemented mutants with a restored genotype. *B. pseudomallei* grown in LB agar containing 50 mM arginine at 37 °C for 24 h under low oxygen conditions (inducers of *arcA* and *arcC* expression). Lane 1, wild type; lane 2, *arcA* mutant; lane 3, *arcC* mutant; lane 4, *arcA* complemented strain; and lane 5, *arcC* complemented strain. M: 100–3000 bp marker ladder.

RT-PCR was used to determine transcript levels of *arcA* and *arcC* for isogenic *B. pseudomallei* strain 153 types I, II and III (Fig. 3A). This demonstrated higher transcript levels of *arcA* and *arcC* for types II and III compared with type I, a finding that is consistent with the proteomic data for these two genes.

We proposed based on homology with these genes in *P. aeruginosa* that *B. pseudomallei arcA* and *arcC* represented genes of an arginine deiminase system. In other bacteria, the ADS is activated by the presence of arginine [12–15] or growth in reduced oxygen concentration [12–15]. We sought evidence that this was also the case for *B. pseudomallei* by determined transcript levels of *B. pseudomallei* strain 153 type I (parental strain) *arcA* and *arcC* in LB broth at pH 7.4 with or without arginine, after incubation in air or under oxygen limitation. We observed that transcript levels were higher for both genes following growth under oxygen limitation compared with growth in air, and that arginine was a strong inducer of gene expression (Fig. 3B). This provided additional evidence that these genes are part of the ADS.

In other bacteria, the ADS is activated by, and provides protection against acid stress [12–15]. We compared transcript levels after growth in LB broth at pH 4.0 versus pH 7.4 following incubation in air or under oxygen limitation. A marked increase was observed in transcript levels of *arcA* and *arcC* at pH 4 compared with pH 7 after incubation in air, findings that are consistent with the published literature.

### 3.3 Construction of *arcA* and *arcC* mutants and complemented strains

*B. pseudomallei* mutants defective in *arcA* or *arcC* were constructed by deletion mutagenesis using a pEXKm5-based allele replacement strategy [8]. Mutants were verified by PCR analysis. The presence of a 578 bp DNA fragment (Fig. 3C, lane 2) and the absence of a 236 bp *oriT* fragment indicated the desired mutant allele, while the wild type BP153 yielded an *arcA* product of 1117 bp (Fig. 3C, lane 1). A similar analysis indicated the presence of a 591 bp DNA fragment in the *arcC* mutant (Fig. 3C, lane 3) and the absence of the 236 bp *oriT* fragment, while the wild type yielded a 915 bp *arcC* product (Fig. 3C, lane 1). These mutants were clearly distinguished from unresolved merodiploids in that the merodiploids yielded DNA fragments indicative of both mutant alleles and integrated pEXKm5 vector sequence typified by *oriT* sequences (data not shown).

Complemented strains were constructed using the same approach. The PCR product size using primers flanking deleted alleles demonstrated that the complemented *arcA* and *arcC* mutant strains had the 578 and 591 bp PCR fragments indicating deletion alleles replaced by wild type sequences which yielded 1117 and 915 bp PCR products, respectively, which were the same as those observed with the parental strain (Fig. 3C, lanes 4 and 5).

RT-PCR analysis was used to verify the deletion of *arcA* and *arcC* genes in the mutants (Fig. 3D). This demonstrated that *arcA* expression was only abolished in the *arcA* mutant (first panel, lane 2), and *arcC* expression only in the *arcC* mutant (second panel, lane 3). The 16S rRNA positive control samples all yielded a 336 bp product (third panel). No amplification was detected in the negative controls (data not shown).

The mutants and complemented strains had the same colony morphotype as the parental strain. Growth curve analysis using starting inoculums of 1 × 10^6^ cfu/ml in 10 ml LB broth at 37 °C incubation with shaking demonstrated that the doubling time of *arcA* and *arcC* mutants was not different from that of wild type (*P* > 0.1). The average doubling times of wild type, *arcA* and *arcC* mutants were 37.2, 37.0 and 38.9 min, respectively. In all strains, log phase started at 6 h and stationary phase at 15 h.

### 3.4 Effect of *arcA* and *arcC* mutations on *B. pseudomallei* survival

**Fig. 4:**
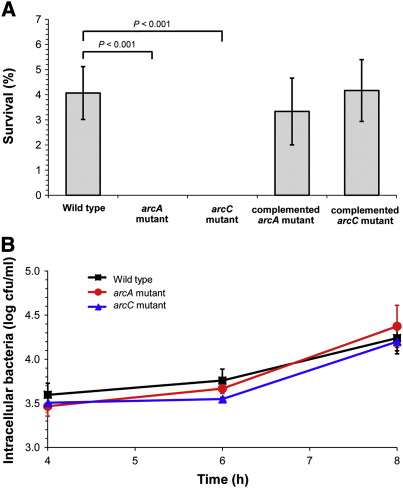
Survival of wild type, *arcA* and *arcC* mutants and complemented strains in acid (A) and in a J774A.1 mouse macrophage cell line (B). (A) *B. pseudomallei* was exposed to LB at pH 3.0 containing 50 mM arginine for 6 h at 37 °C. Bacterial counts were determined by plating serial dilutions onto Columbia agar plates. The results were obtained from triplicate experiments. Data plots are mean ± standard deviations (SD). (B) J774A.1 macrophages were infected with bacteria at a MOI of 10:1. The results represent mean numbers of intracellular bacteria and standard deviations (SD) at 4, 6 and 8 h after infection. Data are from two separate experiments.

Susceptibility to killing by acid was compared between *B. pseudomallei* strain 153 type I (parental) and mutants defective in *arcA* or *arcC* by incubation in LB broth containing 50 mM arginine at pH 3.0 at 37 °C with shaking at 200 rpm for 6 h. Growth of wild type at pH 3.0 led to a 1.5 log reduction from the starting inoculum (1.0 × 10^6^ cfu/ml). This compared with the much more dramatic reduction from a starting inoculum of 1.1 × 10^6^ cfu/ml to no viable bacteria for both the *arcA* and *arcC* mutants (*P* < 0.001) (Fig. 4A). The complemented mutants had a restored phenotype at pH 3.0, with a percentage survival that was comparable to wild type (Fig. 4A).

Having shown that *arcA* and *arcC* transcripts were increased in wild type in response to a low oxygen concentration, we investigated the effect on survival of *arcA* and *arcC* under this condition. Serial dilutions of 1 × 10^8^ cfu/ml of wild type, *arcA* and *arcC* mutants were inoculated onto LB agar adjusted to pH 7.4 and incubated at 37 °C in an AnaeroPack-MicroAero system. Viable bacteria were enumerated and compared to those on plates incubated in air. There was no significant difference observed for survival in low oxygen concentration between wild type, *arcA* or *arcC* mutants at pH 7.4 (*P* > 0.1).

### 3.5 Survival of *arcA* or *arcC* mutants in macrophages

We predicted that a defect in the ADS would not affect the initial bacterial-macrophage cell interaction, but that mutants defective in *arcA* or *arcC* would have a survival disadvantage following uptake. We defined the initial interaction based on the number of cell-associated *B. pseudomallei* after 2 h incubation at a MOI of 10:1. No difference was observed between wild type, *arcA* or *arcC* mutants, with 2.1%, 1.6% and 2.3%, respectively, of the starting inocula becoming cell-associated. Extracellular bacteria were removed at this stage, and the number of intracellular bacteria determined after a total of 4, 6 or 8 h of incubation. The number of bacteria counted at these time points was comparable for wild type and both mutants (Fig. 4B) (*P* > 0.05 for all time points). These data suggest that *arcA* and *arcC* are not required for intracellular survival in a J774A.1 cell line.

## 4 Discussion

The ADS is widely distribute among prokaryotes [6], and has been linked to the ability of bacteria to withstand low pH through the generation of alkali (ammonia). An ADS has been shown to promote survival of *Streptococcus suis*, *Listeria monocytogenes*, *Streptococcus pyogenes* and oral streptococci under acid conditions in vitro [14–17], and to be a virulence factor in an experimental mouse model of *L. monocytogenes* infection [17]. The ADS system of *S. pyogenes* and *S. suis* has also been reported to be involved in adhesion to and invasion of epithelial cells [14,18].

The contribution of the ADS to bacterial fitness and disease pathogenesis in the human host is largely speculative, but includes some potentially important possibilities. For example, it has been proposed that an ADS in *L. monocytogenes* is involved in tolerance to acid during growth in low-pH foods, during successful passage through the stomach, and survival within the macrophage phagosome [17]. An ADS has also been identified in *Salmonella enterica* serovar *typhimurium* that was upregulated in response to low oxygen availability and high osmolarity, conditions that are prevalent in the gut [19]. A striking feature of the highly successful community-associated methicillin-resistant *Staphylococcus aureus* (MRSA) USA300 genome was the horizontal acquisition of a novel mobile genetic element encoding an arginine deiminase pathway (together with an oligopeptide permease system) [20]. The relationship of these genes to the biological fitness of USA300 is unknown, but it has been proposed that they may contribute to bacterial growth and survival. The clearest relationship between a bacterial ADS and humans is in relation to dental caries, the generation of alkali by oral bacteria such as *Streptococcus gordonii* and members of the mutans streptococci being thought to play a key role in plaque pH homeostasis and reduction in risk of caries [21–23].

An ADS has not been described previously for *B. pseudomallei*, but has been studied in *P. aeruginosa* [12,24,25]. In the absence of oxygen and nitrate, *P. aeruginosa* metabolizes arginine via the arginine deiminase pathway, which allows slow growth on rich media [12]. The ADS facilitates survival of *P. aeruginosa* in a pH as low as 3.0 through the degradation of arginine and production of ammonia with a resulting increase in pH [24]. A fascinating observation was made for *P. aeruginosa* isolates recovered over a period of 3 to 5 years from 3 selected patients with cystic fibrosis, in which strains with a mutator or a non-mutator phenotype demonstrated increased expression of the ADS, suggesting a role for long-term adaptation in the human host [26].

The ADS has been reported to be induced by a variety of stimuli in vitro, including the presence of arginine, reduced oxygen tension, acid, temperature shifts and iron limitation [12,13,18,25,27–29]. This is consistent with the findings described here for *B. pseudomallei*. One or more of these stimuli would be predicted to occur during bacterial infection in the human host, including melioidosis where abscess formation is common [1,2]. *B. pseudomallei* is a facultative intracellular bacterium [5,30–33], and has been found in macrophages and multinucleated giant cells in post-mortem tissue [34]. We predicted that the ADS would be involved in survival after uptake by macrophages in vitro, but this did not prove to be the case for the mouse macrophage cell line J774A.1. Furthermore, natural deletion of the ADS operon has been described in a clinical *B. pseudomallei* isolate (strain 708a) that was shown to be virulent in a murine melioidosis model [35], indicating that this system is not essential for bacterial survival or virulence in vivo, but may nonetheless be important in persistence.

Proteins of the ADS were just two of numerous proteins that were differentially expressed in *B. pseudomallei* types II and III compared with type I. This suggests the involvement of a global regulator, leading us to consider the regulation of the ADS operon in other bacteria. Regulation appears to be variable between different bacterial species, and is potentially complex. For example, ArcR is a transcriptional regulator of the ADS in *S. gordonii* [21], *S. suis* [16], and *L. monocytogenes* [17]. Expression of an ADS in *L. monocytogenes* is also modulated by both the alternative stress sigma factor σ^B^ and the central virulence regulator PrfA [17], and multiple two-component systems have been reported to modulate alkali generation in *S. gordonii* in response to environmental stresses [36]. This does not provide us with any indication as to the mechanism for colony morphology switching.

Flagellin encoded by BPSL3319 (*fliC*) was highly expressed in types II and III compared with type I. This finding supports phenotypic evidence of flagella expression reported by us previously, in which electron microscopy revealed that the proportion of flagellate bacteria of isogenic types I, II and III were 10%, 56% and 85%, respectively [4]. Greater motility of live bacteria was also observed by real-time microscopy (RTM-3) for types II or III compared with type I, the proportion of actively motile bacteria being 9%, 77% or 76% of the population observed for isogenic types I, II and III, respectively [4]. The biological effect of increased flagellin expression in the human host is not understood, and has numerous possible explanations including the ability of bacteria to migrate towards nutrients or away from adverse environments, and involvement in interactions with host cells and the host immune response.

Two proteins that were observed to be more highly expressed by type I in comparison to type II or III were possible virulence determinants. The first of these was a protein encoded by BPSS1183 with a putative function similar to a *Pseudomonas syringae* syringomycin biosynthesis enzyme for a phytotoxin or syringomycin product, a known virulence factor that causes the phosphorylation of several membrane polypeptides [37]. This has homology to a newly-described bactobolin antibiotic in *Burkholderia thailandensis* E264 [38]. Also highly expressed in type I compared to type II or III was an oxido-reductase, which has anti-oxidant activity. The expression of these proteins in type I is consistent with our previous finding that type I had greater resistance to killing by hydrogen peroxide in vitro. The complex pattern of protein expression changes observed for isogenic types II and III compared with type I in *B. pseudomallei* strain 153 suggests the simultaneous involvement of numerous bacterial factors in the adaptive process associated with colony morphology switching. Elucidation of just one of these factors provides proof of concept that this was involved in bacterial survival in the presence of acid.

## Figures and Tables

**Table 1:** Primer pairs used in this study.

Primers	Sequence (5′–3′)	Position^a^	Product size (bp)
*arcA*			
*arcA*-F1	CTCAAGTCGGTGTCCATTCC	17–36	280
*arcA*-R1	ACGTTATCCGGCGTGATCT	278–296
*arcA*-F2	AGATCACGCCGGATAACGTGGTACCGTGTTCAGCTTCTG	838–857	317
*arcA*-R2	GTGTACGTGTTGCGGTCGTA	1114–1133
*arcA*-F1	CTCAAGTCGGTGTCCATTCC	17–36	1117
*arcA*-R2	GTGTACGTGTTGCGGTCGTA	1114–1133
*arcA*-comp-F	ACATGTCCCAAGCCATCCCTCA	(− 2)–20	1261
*arcA*-comp-R	GCTCAGTAGTCGACGGGGTCGCG	1237–1259
AF	CACAACCTGCTGACCGAGACCGTG	217–240	220
AR	CGCGAAACAGCGTGAGCACCTT	415–437

*arcC*			
*arc*C-F1	GTATCGTCATCGCATTGGG	5–23	277
*arcC*-R1	AGGTTGCCCATTTCCTGTTC	262–281
*arcC*-F2	GAACAGGAAATGGGGTACCTTGATCGACAAGGATCTGTGC	608–627	334
*arcC*-R2	GAATCCCGTCGACCTTCAC	901–916
*arc*C-F1	GTATCGTCATCGCATTGGG	5–23	915
*arcC*-R2	GAATCCCGTCGACCTTCAC	901–916
*arcC*-comp-F	CCGCACCCCGGGCACCGTTGACACAA	(− 40)–(− 15)	996
*arcC*-comp-R	CGCGCGCCCGGGCGGCATCACCG	934–956
CF	CGCGCAGACGGAAGGGATGAT	228–248	240
CR	CGGCACGACGCGGCGGAACTTG	447–468	

*16S*			
Univ_*16S*_F	TGGCTCAGAACGAACGCTGGCGGC	21-44	336
Univ_*16S*_R	CCCACTGCTGCCTCCCGTAGGAGGAGT	327-356

pEXKm5			
OriT_F	TCCGCTGCATAACCCTGCTTC	598-578	236
OriT_R	CAGCCTCGCAGAGCAGGATTC	368-383

a Positions corresponding to the nucleotide sequence of the indicated genes as annotated on *B. pseudomallei* K96243 chromosome 1 (NCBI Reference Sequence NC 006350) or the pEXKm5 sequence (GenBank accession number GQ200735).

**Table 2:** Summary of altered-protein expression of stationary phase parental *B. pseudomallei* strain 153 type I and isogenic colony variant types II and III.

Functional category	Altered protein	Spot number	Gene designation	Locus ID	Mass	PI	Score	Ratio type II/I or III/I		Protein location/specific function
II	III
**Upregulated proteins**										
Amino acid transport and metabolism	Arginine deiminase	66	*arcA*	BPSL1743	46,422	5.57	359	1.58	2.42	Cytoplasmic
Arginine deiminase	67	*arcA*	BPSL1743	46,422	5.57	241	10.38	21.0	Catalyzes the degradation of arginine to citruline and ammonia
Carbamate kinase	20	*arcC*	BPSL1745	33,507	5.54	129	1.99	3.01	Cytoplasmic
Carbamate kinase	45	*arcC*	BPSL1745	33,507	5.54	130	6.67	10.13	Reversible synthesis of carbamate and ATP from carbamoyl phosphate and ADP
Hypothetical	32	–	BPSL1591	40,435	5.78	208	3.55	2.82	Cytoplasmic
Similar to *Agrobacterium tumefaciens* dehydrogenase
Energy production and conversion	Ferredoxin-NADP(H) reductase	75	*fpr*	BPSL0241	28,983	5.78	155	2.82	2.49	Cytoplasmic
FAD-containing enzyme that catalyzes the reversible electron transfer between NADP(H) and electron carrier proteins such as ferredoxin and flavodoxin
Carbohydrate metabolism	UDP-glucose dehydrogenase	73	*udg*	BPSL2511	50,802	5.34	93	1.53	3.47	May have multiple localization sites
Cell wall/membrane biogenesis
Chaperone	Chaperonin GroEL	29	*groEL*	BPSL2697	57,137	5.13	159	0.51	3.00	Cytoplasmic
60 kDa chaperone family; promotes refolding of misfolded polypeptides especially under stressful conditions
Cell motility	Flagellin	6	*fliC*	BPSL3319	39,233	5.05	106	10.72	15.63	Extracellular
Structural flagella protein

**Down-regulated proteins**										
Lipid transport and metabolism	Succinyl-CoA:3-ketoacid-coenzyme A transferase subunit A	53	*scoA*	BPSL1955	25,373	5.6	155	0.103	0.105	Cytoplasmic
Coenzyme A (CoA) transferases catalyze the reversible transfer of CoA from one carboxylic acid to another
Succinyl-CoA:3-ketoacid-coenzyme A transferase subunit B	54	*scoB*	BPSL1954	22,330	4.7	131	0.133	0.322	Cytoplasmic
Coenzyme A (CoA) transferases catalyze the reversible transfer of CoA from one carboxylic acid to another
Energy production and conversion	Inorganic pyrophosphatase	22	*ppa*	BPSL1021	19,206	5.37	88	0	0	Cytoplasmic
Catalyzes the hydrolysis of pyrophosphate to phosphate
Betaine aldehyde dehydrogenase	61	*aldA*	BPSL1550	50,738	5.67	250	0.490	0.412	Cytoplasmic
Catalyses the conversion of betaine aldehyde to glycine betaine
Amino acid transport and metabolism/	2,3,4,5-tetrahydropyridine-2,6-dicarboxylate N-succinyltransferase	74	–	BPSL2169	29,660	5.68	181	0.136	0.386	Cytoplasmic
Catalyzes the formation of N-succinyl-2-amino-6-ketopimelate from succinyl-CoA and tetrahydrodipicolinate in the lysine biosynthetic pathway
Inorganic ion transport and metabolism	Sulfurtransferase	49	*sseA*	BPSS1766	31,119	5.98	127	0.345	0.170	Cytoplasmic Cyanide detoxification
Catalyzes thiosulfate and cyanide to sulfite and thiocyanate
Secondary metabolites biosynthesis, transport and catabolism	Non-ribosomally encoded peptide/polyketide synthase	35	*phyH*	BPSS1183	35,611	5.77	102	0.725	0.142	Cytoplasmic membrane
*Pseudomonas syringae* syringomycin biosynthesis enzyme or *B. thailandensis* bactobolin
Posttranslational modification, protein turnover	Oxido-reductase	62	–	BPSL2748	23,904	5.75	95	0.085	0.081	Cytoplasmic
Peroxidase
Antioxidant proteins
Multifunctional	Acetoacetyl-CoA reductase	72	*phbB*	BPSS1916	26,583	6.3	190	0.763	0.105	Cytoplasmic
Synthesizes polyhydroxybutyrate (PHB) from acetyl coenzyme A (acetyl-CoA) in *Ralstonia eutropha*.
Unknown	Hypothetical protein	71	–	BPSL1549	23,384	5.14	66	0.417	0.091	Unknown

Protein spots were separated using a pH range 4–7 and examined using 2D Image master software. Only those proteins with a reproducible change in spot intensity volume between types I versus II or I versus III colony variants of ≥ 1.5-fold in two independent experiments are reported. Functional groups were identified based on COG functional category (http://www.ncbi.nlm.nih.gov). PSORTb was used to predict protein localization (http://www.psort.org/psortb).
